# BioN∅T: A searchable database of biomedical negated sentences

**DOI:** 10.1186/1471-2105-12-420

**Published:** 2011-10-27

**Authors:** Shashank Agarwal, Hong Yu, Issac Kohane

**Affiliations:** 1 Medical Informatics, College of Engineering and Applied Sciences, University of Wisconsin-Milwaukee, 3200 N. Cramer St., Milwaukee WI 53201-0784, USA; 2 Department of Computer Science and Electrical Engineering, College of Engineering and Applied Sciences, University of Wisconsin-Milwaukee, 3200 N. Cramer St., Milwaukee WI 53201-0784, USA; 3 Department of Health Sciences, College of Health Science, University of Wisconsin-Milwaukee, 2400 E. Hartford Ave., Milwaukee WI 53211, USA; 4 Children's Hospital Informatics Program, Children's Hospital, 300 Longwood Ave., Enders-6, Boston MA 02115, USA

## Abstract

**Background:**

Negated biomedical events are often ignored by text-mining applications; however, such events carry scientific significance. We report on the development of BioN∅T, a database of negated sentences that can be used to extract such negated events.

**Description:**

Currently BioN∅T incorporates ≈32 million negated sentences, extracted from over 336 million biomedical sentences from three resources: ≈2 million full-text biomedical articles in Elsevier and the PubMed Central, as well as ≈20 million abstracts in PubMed. We evaluated BioN∅T on three important genetic disorders: autism, Alzheimer's disease and Parkinson's disease, and found that BioN∅T is able to capture negated events that may be ignored by experts.

**Conclusions:**

The BioN∅T database can be a useful resource for biomedical researchers. BioN∅T is freely available at http://bionot.askhermes.org/. In future work, we will develop semantic web related technologies to enrich BioN∅T.

## Background

In the biomedical domain, a large amount of published literature is available in electronic format, spurring the development of several text-mining applications that can process the available literature to automatically extract information such as protein-protein interaction and gene-disease association. Unfortunately, the text mining community tends to focus on positive events only. Many text-mining applications either ignore sentences containing negation or do not process negation at all, a situation that could lead to negated events being identified as positive events. We argue that negated events provide valuable information and may help researchers formulate research hypotheses.

A use case for extracting negated events can be seen in the case of genomic incidentalomes [1]. As genomic medicine develops to offer genome-level screening tests, it is important to identify genes that were earlier believed to be associated with a disease, but eventually were found not to be associated with the disease. Such genes should be removed from the array of genomic tests a patient undergoes since failure to do so will result in the patient being subjected to unnecessary tests, causing additional morbidity, and hence, increasing the cost of genomic medicine substantially. Finding reported instances of a gene not being associated with a disease is difficult, which is why our goal in this study is to develop a text mining application that can identify such negated relations.

In this study we attempt to fill the gap created due to the absence of text mining applications that extract negated events. Our long-term goal is to expand the existing BioN∅T system to identify biomedical named entities (e.g., gene and protein names), and therefore enable BioN∅T to capture negative relations between named entities. Here we report the development of a database called BioN∅T, which contains negated sentences from three sources: abstracts of articles indexed by PubMed, full-text of articles in the PubMed Central Open Access Subset, and full-text of articles published by Elsevier publisher. We have indexed the sentences in BioN∅T and made them available online through a search engine, available at http://bionot.askhermes.org/.

### Related works

Detection of negation in biomedical literature is an important task. As evidence, the BioNLP'09 Shared Task on Event Extracted included negation detection as one of the tasks. Several text mining applications exploring negation identification have been developed.

In the clinical domain, rule-based approaches have been developed for negation detection. For example, Chapman et al. [2] developed the NegEx system to identify negation of target findings and diseases in narrative medical reports. The current version of NegEx uses 272 rules, which are matched by using regular expression patterns. The reported recall of the system was 95.93%, precision was 93.27%, and accuracy was 97.73%. A similar system, Negfinder, was developed to identify negated concepts in medical narratives [3]. The system first identifies negation markers in the sentence by using regular expression patterns. These words are then passed to a parser that uses a single-token look-ahead strategy to identify negated concepts. The reported recall and precision of the system were 95.27% and 97.67%, respectively. Along the same lines, Elkin et al. [4] developed a system to identify the negation of concepts in electronic medical records. The system was built by identifying textual cues for negation in 41 clinical documents. The reported recall and precision of the system was 97.2% and 91.2%, respectively. A hybrid approach that classifies negations in radiology reports based on the syntactic categories of the negation signal and negation patterns was developed by Huang and Lowe [5]. Thirty radiology reports were manually inspected to develop the classifier and the classifier was validated on a set of 470 radiology reports. Evaluation was conducted on 120 radiology reports and the reported recall and precision were 92.6% and 98.6%, respectively.

Supervised machine-learning approaches have also been developed for negation detection. Averbuch et al. developed an algorithm to automatically learn negative context patterns in medical narratives [6]. The algorithm uses information gain to learn negative context patterns. Aramaki et al. developed a negative event recognition module for their medical text summarization system [7]. The module was based on a supervised machine-learning algorithm, Support Vector Machines, which uses syntactic information to detect negation.

In the genomics domain, a rule-based system was developed by Sanchez-Graillet and Poesio to detect negated protein-protein interactions in the biomedical literature [8]. The system was built using a full dependency parser. Hand-crafted rules were then used to detect negated protein-protein interaction. An example rule reads as follows: if cue verb, such as 'interact,' is an object of 'fail,' 'Protein A' is subject of fail, and 'Protein B' is object of interact, then there is no interaction between 'Protein A' and 'Protein B.' Evaluation was conducted on 50 biomedical articles and the best recall and precision reported were 66.27% and 89.15%, respectively.

If a negation is reported in a sentence, it might not apply to the entire sentence. For example, in the sentence, *''While there was no difference in overall growth between BRCA1+ and BRCA1 wt cells, BRCA1+ cells showed a marked reduction in survival following STS treatment.'' *the negation marker *'no' *negates the observation *'difference in overall growth between BRCA1+ and BRCA1 wt cells following STS treatment.' *The observation *'BRCA1+ cells showed a marked reduction in survival following STS treatment' *is positive and not modified by the negation marker. Hence, it is important to identify the scope of negation as well. The following studies identify the scope of negation in a sentence.

Morante and Daelemans [9] developed a two-phase approach to detect the scope of negation in biomedical literature. In the first phase, negation cues were identified by a set of classifiers. In the second phase, another set of classifiers was used to detect the scope of the negation. The system performed better than the baseline in identifying negation signals in text and the scope of negation. The percentages of correct scope for abstract, full-text and clinical articles were 66.07%, 41.00% and 70.75%, respectively.

We developed a negation scope detection algorithm called NegScope previously [10]. NegScope was developed by training supervised machine-learning algorithm conditional random field (CRF) [11] using words and parts of speech as features. The CRF models were trained on the BioScope dataset [12], which contains more than 20,000 manually annotated sentences from clinical notes and published biological articles. In each sentence, the scope of negation and hedging has been annotated. On evaluating NegScope, we found that it predicted the negation status of a biological sentence with 99.86 % accuracy and 96.5 % F1-score. The F1-score was calculated as the harmonic mean of precision and recall. NegScope correctly identified the scope of negation in 81% of biological sentences. To our knowledge, NegScope is the only open-source package that can detect scope of negation in biological text and one of two open-source packages for clinical notes. Moreover, we found that it performed better than other negation or negation scope detection algorithms when tested on biological sentences [10].

BioContrasts [13] was developed to detect and search contrastive relations between proteins. In this system, contrastive information was extracted using manually curated patterns such as 'A but not B,' where A and B were restricted to protein names from Swiss-Prot entries. A total of 41,471 contrast relations were identified by the system from 2.5 million Medline abstracts.

## Construction and content

### Source of sentences for BioN∅T

As mentioned earlier, BioN∅T is a searchable database of negated biomedical sentences. We obtained these sentences from three sources - (1) abstracts of Medline articles (≈ 19 million abstracts; ≈ 101 million sentences), (2) full-text of PubMed Central Open Access Subset (≈ 167,000 articles; ≈ 20 million sentences) and (3) full-text of articles published by Elsevier publisher (≈1.9 million articles; ≈ 215 million sentences). We split articles for sentences using the NaCTeM sentence splitter [14].

### Using NegScope to detect scope of negation

Many text-mining applications make use of sentences to extract information from literature. These sentences often contain multiple entities. If a negation is reported in such sentences, it might not apply to all entities in the sentence. To identify the negated entities, it is important to identify the scope of negation. As mentioned in the Related Works, the NegScope algorithm that we developed can identify the scope of negation; hence, we used it to build BioN∅T.

### BioN∅T database and search engine

To prepare the BioN∅T database, we tagged each extracted sentence. The previous and following sentences were stored to provide contextual information. For the search engine, we indexed all negated sentences and the sentences preceding and following the negated sentence using the open-source Apache Lucene package [15]. The preceding and following sentences are displayed along with the search results. We also indexed the negation scope in the sentence. When the user enters a query, we retrieve relevant sentences based on the terms in the query. Here, a term is an individual word in the query. When searching for negated events, we applied the following heuristic - if a single term is used, it can be present anywhere in the negated sentence; if more than one term is used, all terms should appear in the negated sentence and at least one of those terms should appear within the scope of negation. Note that since the scope of negation is a part of the sentence, terms appearing in the scope of negation appear in the sentence as well.

### Autism, Alzheimer's Disease and Parkinson's Disease Use Case

As described in the Background section, in case of incidentalomes, it is important to identify genes that were earlier believed to be associated with a disease, but eventually were found not to be associated with the disease. Sometimes literature is published indicating that the association is not held in certain circumstances. With BioN∅T, our goal is to develop a searchable database that can be used by researchers to identify such negated relationships. To test the utility of BioN∅T, we evaluated it on the detection of knowledge of three important genetic diseases: Autism, Alzheimer's disease, and Parkinson's disease. Several genes have been thought to be associated with the manifestation of these diseases. We consulted published reviews to identify genes thought to be associated with these diseases and found 26 putative genes for autism (see Table Two in [16]), 10 for Alzheimer's disease (see Table Two in [17]), and 6 for Parkinson's disease (see Table Two in[18]). Using BioN∅T, we searched for each disease and its putative gene as query. We also searched the index by replacing the disease name with related keywords; for example, autism was replaced with keywords *'ASD' *(Autism Spectrum Disorder) and *'autistic.' *We manually analyzed the sentences that were returned for autism to analyze the errors in our system.

## Utility

To build the BioN∅T database, we analyzed a total of 336 million sentences, out of which 32 million sentences had negation; hence, 9.53% of all sentences contained negation in them (Table 1). It should be noted that these sentences contained some form of negation, and do not necessarily indicate negation between biomedical entities.

**Table 1:** Negated sentences statistics

	PMC	PubMed	Elsevier	TOTAL
Title sentences	167,691	18,974,626	1,914,879	21,057,196
Title negated	6105	414,809	19,430	440,344
Title %	3.64	2.19	1.01	2.09
Abstract sentences	1,060,652	82,320,574	8,970,587	92,351,813
Abstract negated	114,772	9,298,962	702,280	10,116,014
Abstract %	10.82	11.30	7.83	10.95
Full-text sentences	18,920,031	0	204,459,184	223,379,215
Full-text negated	2,360,129	0	19,180,949	21,541,078
Full-text %	12.47	-	9.38	9.64
Total sentences	20,148,374	101,295,200	215,344,650	336,788,224
Total negated	2,481,006	9,713,771	19,902,659	32,097,436
Total %	12.31	9.56	9.24	9.53

Number and proportion of negated sentences in PubMed Central Open Access Subset (PMC), PubMed and Elsevier articles.

We searched BioN∅T for negated sentences containing a potential autism, Alzheimer's disease or Parkinson's disease-related gene (list of genes obtained from [16-18]) and the disease name. We found negated relation evidence for 12 out of 26 autism-related genes (Table 2), 8 out of 10 Alzheimer's disease-related genes (Table 3), and 3 out of 6 Parkinson's disease-related genes (Table 4).

**Table 2:** Negated genes for Autism

Gene	Sentence
EN2	Do the genetic data add to the overall hypothesized neurophysiological mechanism, or are the data less focused? In the end, RELN, 5 HTT and EN2 may not be major genes in the etiology of autism, either singly or in concert, but they are important models for pointing out the difficulties in these studies so that advances in understanding the genetic and developmental basis of autism can be attained.
GRIK2	After applying Bonferroni correction, these results were no longer statistically significant. The global 2 -test or association regarding the number of haplotypes (H) for 1 degree of freedom (d.f.) for haplotype transmission did not reveal an association between the GRIK2 locus and ASD (2 = 19.355, d.f. = 13). We also carried out the bootstrap significance test using 100,000 bootstrap samples.
SLC25A12	Furthermore, a strong association of autism with SNPs within SLC25A12, a gene encoding the mitochondrial aspartate/glutamate carrier (AGC1), has been demonstrated, suggesting the potential etiological role of AGC1 in autism (Ramoz et al., 2004; Segurado et al., 2005). However, recent two studies using large samples did not confirm the association of SLC25A12 gene and autism, suggesting that the SLC25A12 gene is not a major contributor to genetic susceptibility of autism (Blasi et al., 2006; Rabionet et al., 2006). Second, it has been reported that blood levels of glutamate are altered in patients with autism (Rolf et al., 1993; Moreno-Fuenmayor et al., 1996; Aldred et al., 2003).
OXTR	We observed AEI in OXTR. The variation in AEI was driven, in part, by a SNP in intron 3 of OXTR (rs237897; p = 0.0265). rs237897 was not associated with autism in our sample. The addition of hormones did not appear to alter AEI significantly from the baseline.
SHANK3	In addition, our results also reinforce the need for the detailed LD mapping, mutation screening and CNV analysis of SHANK3 in different population or other neurodevelopmental disorders. The present study did not find strong evidence of SHANK3 polymorphisms and autism or identify any described non-synonymous mutations in our cohort. These might indicate that SHANK3 doesn't represent a major susceptibility gene for autism in the autism families ascertained from Chinese Han population.
SLC6A4	Based on these results, it appears unlikely that SLC6A4 play a significant role in the genetic predisposition to autism. In this study, no evidence was provided for an association between the SLC6A4 locus and autism in the Chinese Han trios. What reasons might be considered for the differences?
CADPS2	Despite positional, functional, and expression data supporting the role of CADPS2 as a candidate gene for autism, we were unable to identify any mutations in or around the coding regions that co-segregate with the disorder in 90 families multiplex for autism. The A297T mutation found in autism family AU427 does not occur in a conserved region of the gene (the amino acid at codon 297 differs between human CADPS2 and mouse cadps2), and does not occur within any known functional domains of the protein, and thus is unlikely to be functionally relevant. Human CADPS and CADPS2 were cloned from a brain cDNA library using the yeast two hybrid system with the C terminus of dystrophin as bait.
NLGN3	A family-based association study for rs2290488 in 101 trios did not reveal association of this polymorphism with autistic disorders on high functioning level. We conclude that there is no evidence for an involvement of NLGN3 and NLGN4X genetic variants with autism spectrum disorder on high functioning level in our study group.
GABRB3	Serotonin transporter (5 -HTT) and gamma-aminobutyric acid receptor subunit beta3 (GABRB3) gene polymorphisms are not associated with autism in the IMGSA families. The International Molecular Genetic Study of Autism Consortium.
MECP2	However, they were unable to confirm this change in mRNA. Vourc ? h et al. (50) failed to identify mutations in the MeCP2 coding sequence in a sample of 59 patients with autism, only 17 of which were females. Both of the mutations described in the current study have been noted in classic RTT patients.
UBE3A	A population-based study showed a high rate of ASD in AS (38). But, a mutation was not identified in the UBE3A putative promoter or coding region in 10 idiopathic ASD patients (39). Lack of expression of the maternally expressed UBE3A gene in the brain is thought to be the cause of AS.
RELN	Furthermore, analysis of a previously reported triplet repeat polymorphism and intragenic single nucleotide polymorphisms, using the transmission disequilibrium test, provided no evidence for association with autism in IMGSAC and German singleton families. The analysis of RELN suggests that it probably does not play a major role in autism aetiology, although further analysis of several missense mutations is warranted in additional affected individuals.

Sample sentences indicating absence of relationship between a putative gene and autism. The sentences preceding and following the negation sentence are also included to provide context.

**Table 3:** Negated sentences for Alzheimer's disease

Gene	Sentence
ACE	However these findings have not been confirmed by other reports (2,5,15,18,20). Among Italian studies, <negation>no association has been reported between ACE I/D polymorphism</negation> and AD (14,18,20), even if Palumbo et al. showed an increased frequency of D allele in subjects with cognitive impairment (14). In the present study, we investigated the role of ACE I/D polymorphism in a group of sAD patients.
CH25H	From our results we conclude that the functional SNPs within LIPA and FLJ22476 are not associated with AD and therefore are not involved in pathogenetic mechanism leading to AD. Our data further do not support a relevant implication of both CH25H promoter polymorphisms and AD.
CST3	There was no interaction between CST3 with age or APOE. Our findings do not support a role of CST3 gene in Italian sporadic AD.
GAB2	Next, we explored GAB2 rs2373115 SNP singlelocus association using different genetic models and comparing AD versus controls or NNE controls. No evidence of association with AD was observed for this GAB2 marker (p > 0.17). To evaluate GAB2-APOE genegene interactions, we stratified our series according to APOE genotype and case-control status, in accordance with the original studies.
MAPT	CONCLUSIONS: No evidence was found for an association of the non-synonymous polymorphism (Q7R) in STH and Alzheimer's disease. This finding is in line with earlier studies showing no association between MAPT and Alzheimer 's disease.
PRNP	No significant association was found for the PRNP polymorphism in AD compared to controls either in Probable or in Definite AD series even after stratification for APOE polymorphism. This study does not support a role of PRNP polymorphism as a susceptibility factor for AD.
SORL1	Testing for association using dense SNPs in the SORL1 gene did not reveal significant association with AD, or with cognitive function when adjusting for multiple testing. In conclusion, our data do not support the hypothesis that genetic variants in SORL1 are related to the risk of AD.
TF	No linkage disequilibrium between the BCHE K and TF C2 was observed either in both the AD patients and controls (P > 0.1). In conclusion, neither the BCHE K nor the TF C2 confers a risk for AD.

Sample sentences indicating absence of relationship between a putative gene and Alzheimer's Disease. The sentences preceding and following the negation sentence are also included to provide context.

**Table 4:** Negated sentences for Parkinson's disease

Gene	Sentence
PINK1	The phenotypic spectrum associated with PINK1-positive patients may be wider than previously reported. Polymorphisms of PINK1 do not appear to modulate risk of PD in our population.
UCHL1	UCHL1 genotyping is performed routinely in research settings; however, a UCHL1 laboratory test is not commercially available at this time. Given the lack of conclusive evidence supporting a strong association between UCHL1 polymorphisms and Parkinson's disease, it seems unlikely that UCHL1 population testing will be undertaken in the near future. The Venice criteria were developed by the Human Genome Epidemiology Network (HuGENet) Working Group to provide guidance in assessing the cumulative epidemiologic evidence of genetic association studies (104).
LRRK2	No association could be demonstrated. We have therefore no evidence for the existence of a common variant in LRRK2 that has a strong influence on Parkinson's disease risk.

Sample sentences indicating absence of relationship between a putative gene and Parkinson's Disease. The sentences preceding and following the negation sentence are also included to provide context.

We manually analyzed the sentences that were returned for autism. A total of 141 sentences for 20 genes were obtained when we searched the BioN∅T database with autism and genes thought to be associated with autism. NegScope correctly identified negation in 137 out of 141 sentences, which was consistent with the 97% F1-score observed for negation cue detection with NegScope. On the other hand, we found that 81 out of the 137 sentences did not establish a negated relation between the designated gene and disease. We therefore consider that a total of 85 sentences (81 + 4) were false positives. The remaining 56 sentences were considered to be true positives. Our results show that the precision for detecting a negated relation between a gene and a disease is 40%. The 56 true positive sentences show negated relations for 12 genes (out of the 20 genes returned by BioN∅T).

We further analyzed the 85 false positive sentences and found that they can be grouped into three categories: (1) No negated relation (31 sentences), (2) Ambiguous negated relation (17 sentences), and (3) Ambiguous terms (37 sentences) (see false positive sentences for categories (1), (2) and (3) in Additional File 1 Additional File 2 and Additional File 3 respectively). The following list shows example false positive sentences (a), (b) and (c) for categories (1), (2) and (3), respectively -

(a) Because deletions encompassing OXTR have not been observed in other studies characterizing structural variation in autism (58,64,65) such events appear to be rare.

(b) A scan of the NRXN1 coding sequence in a cohort of ASD subjects, relative to non-ASD controls, revealed that amino acid alterations in neurexin 1 are not present at high frequency in ASD.

(c) None of them met, or had ever met, the diagnostic criteria for autism.

The four sentences for which NegScope did not correctly identify negation were classified as category 1 false positives. In another four category 1 false positive sentences, the sentence boundaries were not correctly identified, leading to a false positive relation. All of the category 3 false positives were caused due to the same gene, *MET*, which is also a common English word.

## Discussion

In this study, we report the development of BioN∅T, a publicly available database of 32 million negated sentences taken from three major literature resources: PubMed, PubMed Central, and Elsevier. BioN∅T is currently the only database available that reports negated events reported in biomedical literature. Our study found that almost 10% of sentences published in biomedical literature incorporated negated information. The statistics indicate that negated events are abundant in biomedical literature and therefore BioN∅T can be an important resource for biomedical scientists.

After evaluating negated sentences for autism, Alzheimer's disease, and Parkinson's disease, we found many genes that are thought to be relevant by experts incorporate biomedical evidences suggesting the opposite.

Despite its utility, BioN∅T has several limitations. Although extensive, it is not comprehensive as there are several full-text articles that were not analyzed by BioN∅T. BioN∅T relies on NegScope to identify and mark negation scope; hence, errors in NegScope's predictions could result in certain negated cases being missed by BioN∅T. Moreover, we used the heuristic that an event is negated if all entities in the query are present in the same sentence and at least one of them is within the scope of negation. However, given the nature of discourse, this situation may not always be true. For example, in the following sentence, the negation scope is marked in boldface, and it can be seen that the genes *FMR1*, *TSC1*, *TSC2*, *NF1 *and *MECP2 *are not negated; however, BioN∅T marked the association between these genes and autism as negative - *To date, genome scans, linkage and association studies, chromosomal rearrangement analyses and mutation screenings have identified: (i) genomic regions likely to contain autism susceptibility loci on human chromosomes 1 q, 2 q, 5 q, 6 q, 7 q, 13 q, 15 q, 17 q, 22 q, Xp and Xq; (ii) genes whose mutations represent a rare cause of non-syndromic autism (NLGN3 and NLGN4) or yield syndromic autism (FMR1, TSC1, TSC2, NF1 and MECP2); and (iii) candidate vulnerability genes, with potential common variants enhancing risk but **not causing autism per se ***(Table 1). Finally, BioN∅T is not aware of the semantic category of the target entities, which can lead to false positives. For example, gene *MET *is thought to be associated with autism because several irrelevant sentences have the word 'met' in them but it is not used as a gene name.

Our results show that a long way still remains before negated events can be incorporated for genetic diagnosis. Additional semantic information may benefit the task, including complete or incomplete penetrance, gene expression, and molecular functions.

### Future work

We plan to address some of the above mentioned limitations as future work. First, we plan to mark the semantic categories of words in the negated sentences. Specifically, we plan to mark entities such as genes, diseases, drugs, cells, chemicals, species and other biomedical entities within these sentences. This approach would help avoid false positives when one of the target entities is also a common English word or when an acronym is ambiguous. Marking semantic information would also help to identify cases when synonyms of entities might have been used. We will also explore heuristics that can better identify if the relationship between two entities is negated or not.

## Conclusions

Although often ignored, negated sentences contain valuable information. To capture this information, we have automatically identified negated sentences from various published repositories and built a database of negated sentences called BioN∅T. Currently, BioN∅T comprises 32 million sentences. To make the identified sentences publicly available, we have built a search engine that is available online. We showed that our system can be used to find negated relation between genes and diseases by identifying negated relation between three genetic disorders: autism, Alzheimer's disease and Parkinson's disease, and genes thought to be associated with these disorders.

Besides identifying negated gene-disease relationship, our system can be used to identify published negated events between chemicals, drugs, diseases, cells, and other biomedical entities. Although currently our system is currently text-based, in the future, we plan to identify various biomedical entities and normalize them to improve the performance of this system.

## Availability and requirements

BioN∅T can be freely accessed online at http://bionot.askhermes.org from any modern web-browser.

## Authors' contributions

SA developed the system and conducted evaluation of the BioN∅T system. HY and IK provided guidance. All authors read and approved the final manuscript.

## Supplementary Material

Additional file 1 **No negated relation between gene and disease**. This file lists the false positive associations caused because a negated association did not exist between the gene and disease. Click here for file

Additional file 2 **Ambiguous negated association between the gene and disease**. This file lists the false positive associations caused because the negated association between the gene and disease was ambiguous. Click here for file

Additional file 3 **Ambiguous terms**. This file lists the false positive associations caused because either the gene name or the disease name was ambiguous. Click here for file
