# Supplementary material for: BioN∅T: A searchable database of biomedical negated sentences
Source: BMC Bioinformatics. 2011 Oct 27;12:420. doi: 10.1186/1471-2105-12-420 (PMC3225379; doi:10.1186/1471-2105-12-420)
Supplement: Additional file 1 — No negated relation between gene and disease. This file lists the false positive associations caused because a negated association did not exist between the gene and disease. [file 1471-2105-12-420-S1.DOCX]

Additional file 1: No negated relation between gene and disease

| Count | Gene | Sentence | Notes |
| --- | --- | --- | --- |
| 1 | SLC25A12 | Evidence for a genetic association between autism and two single nucleotide polymorphisms ( SNPs ) , rs2056202 and rs2292813 , in the mitochondrial aspartate / glutamate carrier ( SLC25A12 ) gene led us to ask <negation>whether any of the four previously identified familial traits in autism spectrum disorders ( ASD ) varied by these SNPs</negation> . | Scope error |
| 2 | OXTR | <negation>Because deletions encompassing OXTR have not been observed in other studies characterizing structural variation in autism [ 58,64,65 ] such events appear to be rare</negation> . |  |
| 3 | OXTR | Detailed DNA methylation analysis of the promoter region of OXTR , prompted by the observation that the proband 's autistic brother did <negation>not carry the novel deletion , identified a single CpG dinucleotide within a known OXTR control region</negation> [ 56 ] that , independently , showed a statistically significant increase in methylation in PBMCs and the temporal cortex tissue of autism cases compared to controls . |  |
| 4 | OXTR | Genomic rearrangements of note included a 12.5 Mb deletion within 2q24.1 -2q24.3 , a region previously linked to autism [ 45,46 ] and containing 41 known genes ; a 4.6 Mb duplication and a 3.4 Mb deletion within 15 q11 -13 that contains a complex arrangement of CNVs and has previously been associated with autism by numerous genetic linkage [ 47-49 ] and genome copy number studies [ 12-23 ] ; and a 0.7 Mb deletion in 3p25.3 that contains 5 genes : Lim and cysteine-rich domains 1 , LMCD1 ( MIM accession <negation>no</negation> . : 604859 ) ; C3 orf32 ; Caveolin 3 , CAV3 ( MIM accession <negation>no</negation> . : 601253 ) ; oxytocin receptor , OXTR ( MIM accession <negation>no</negation> . : 167055 ) ; and RAD18 ( MIM accession <negation>no</negation> . : 605256 ) ( Figure 1 ) . | Scope error |
| 5 | SHANK3 | Such crosstalk could also contribute to psychiatric disorders , since a Shank3 deletion mutant <negation>lacking the Homer binding site has been identified in autism</negation> [ 18 ] . |  |
| 6 | SHANK3 | Five of seven cases reported to date carry deletions ( 142 kb to 4.36 Mb in size ) , of the terminal 22 q13 region encompassing SHANK3 , whereas the other two have a frameshift ( E409X ) and a missense mutation ( Q321R ) .40 41 Furthermore , rare non-synonymous variations present in the ASD group , but <negation>not in the control sample</negation> , were also found in both studies.4 0 41 However , these variations were inherited from healthy parents and sometimes were also present in unaffected siblings , suggesting that they may confer vulnerability <negation>rather than play a dominant role in ASD pathogenesis</negation> . | Sentence break error |
| 7 | SLC6A4 | <negation>Some evidence for genetic linkage has been observed between autism and markers covering the 17q11.2 region</negation> and the SLC6A4 gene ( International Molecular Genetic Study of Autism Consortium [ IMGSAC ] 2001 ; Cantor et al 2005 ; McCauley et al 2004 ; Stone et al 2004 ; Sutcliffe et al 2005 ; Yonan et al 2003 ) . | Scope error |
| 8 | SLC6A4 | We found <negation>no transmission disequilibrium of any of the SLC6A4 variants in ASD</negation> . |  |
| 9 | FMR1 | 1 ) gene2 which leads to transcriptional silencing of FMR1 and thus , <negation>absence or significant reduction of the FMR1 protein ( FMRP ) .3</negation> Because FMR1 is located on the X chromosome , females with a full mutation are more mildly affected than males , due to production of FMRP from the normal FMR1 allele on the non-mutated X chromosome . FMRP is an RNA binding protein which modulates dendritic maturation and synaptic plasticity through mechanisms including inhibition of group 1 metabotropic glutamate receptor (mGluR1 and mGluR5) mediated mRNA translation in dendrites.4?7 Numerous expected consequences of excessive activation of mGluR mediated dendritic protein synthesis due to loss of inhibitory control by FMRP are found in the fmr1 knockout mouse, including enhanced mGluR activated hippocampal8 and cerebellar9 long term depression (LTD), reduction of synaptic AMPA receptors,10 immature appearing elongated dendritic processes,11 12 and abnormal epileptiform discharges.13 Further, many phenotypic features of FXS are predicted effects that would occur in a setting of enhancement of mGluR mediated processes, including seizures, epileptic abnormalities on electroencephalograms (EEGs), cognitive problems, strabismus, enhanced anxiety, perseverative behaviours, coordination problems, hypersensitivity to tactile stimuli, and even loose stools.10 | Sentence break error |
| 10, 11, 12, 13, 14 | FMR1, NLGN3, TSC2, MECP2, TSC1 | To date , genome scans , linkage and association studies , chromosomal rearrangement analyses and mutation screenings have identified : ( i ) genomic regions likely to contain autism susceptibility loci on human chromosomes 1 q , 2 q , 5 q , 6 q , 7 q , 13 q , 15 q , 17 q , 22 q , Xp and Xq ; ( ii ) genes whose mutations represent a rare cause of non-syndromic autism ( NLGN3 and NLGN4 ) or yield syndromic autism ( FMR1 , TSC1 , TSC2 , NF1 and MECP2 ) ; and ( iii ) candidate vulnerability genes , with potential common variants enhancing risk but <negation>not causing autism per se ( Table 1 )</negation> . |  |
| 15 | FMR1 | <negation>The relations between autism and gene FMR1 , which causes FXS , are not limited to the complete mutation</negation> -some extremely interesting correlations between autism and the premutation of this gene are also being found . |  |
| 16, 17 | NLGN3, MECP2 | Methyl-CpG-binding protein 2 ( MeCP2 ) is a transcriptional repressor that binds to methylated CpG dinucleotides generally located at gene promoters and recruits HDAC1 and other proteins involved in chromatin repression.1 1 De novo mutations of the MECP2 gene located on chromosome Xq28 occur in 80% of female patients with Rett syndrome , a pervasive developmental disorder generally characterised by regression , autism , microcephaly , stereotyped behaviours , epilepsy and breathing problems , whereas in males , mutations are generally lethal.1 0 11 Some mutations can also result in asymptomatic phenotypes , mild MR and verbal Rett variants.1 1 The clinical phenotype depends upon the type of mutation and the specific X-inactivation pattern , which is highly skewed in the presence of mutations affecting X-linked genes , such as NLGN3 and MECP2 , but is <negation>not significantly skewed in ASD families overall.4</negation> 7 Several groups have screened the MECP2 gene for mutations in patients with non-syndromic ASD (table 4). | Sentence break error |
| 18 | NLGN3 | Additionally , the NLGN3 transcript was present in two isoforms ( with and <negation>without exon 7 ) in nine of 10 autistic females</negation> and in 30 non-autistic subjects , including parents of the autistic female having only the complete transcript with exon 7 , and from the whole brain of a control . |  |
| 19 | NLGN4X | Although <negation>cognitive effects of reduced NLGN4X dosage have not been described in female carriers of these mutations</negation> , autistic features have been described as part of the 45 , X TS neurocognitive phenotype [ 46 ] , and three females with autism and deletions of distal Xp encompassing NLGN4X have been reported [ 47 ] . |  |
| 20 | PTEN | To determine whether germline mutations of PTEN may lead to different phenotypes , we screened all the nine exons of the PTEN gene in 40 patients with neurodevelopmental disorders , with or <negation>without features of autism spectrum disorder</negation> , associated with macrocephaly . |  |
| 21 | TSC2 | Despite the caveat that observed copy number gains need <negation>not map to the wild-type locus</negation> , known ASD genes including TSC2 [ 45 ] and RAI1 [ 44 ] , [ 46 ] within the Potocki-Lupski Syndrome critical interval were amongst the 159 loci observed in at least one AGRE case , but <negation>no CHOP / NINDS controls ( Table S4 )</negation> . |  |
| 22 | MECP2 | In a second study , using a hypothesis-driven approach , Samaco et al ( 2004 ) tested if Rett syndrome , autism , and other neurodevelopmental disorders <negation>without MeCP2 mutations show altered MeCP2 expression</negation> . |  |
| 23 | MECP2 | Shibayama and collaborators ( 2004 ) [ 63 ] described two autistic subjects with 3 -UTR MeCP2 variants , while Samaco and co-workers ( 2004 ) found mainly reduced MeCP2 levels in postmortem brain samples from 6 patients with autism spectrum disorders ( 2 had increased MeCP2 ) and <negation>no MeCP2 mutations</negation> [ 64 ] . |  |
| 24 | MECP2 | The aims of this study were to compare the early and subsequent clinical courses of female subjects with Rett syndrome categorised by whether or <negation>not a diagnosis of autism had been proposed before Rett syndrome had been diagnosed</negation> and compare the spectrum of methyl-CpG binding protein 2 ( MECP2 ) mutations identified among the two groups . |  |
| 25 | MECP2 | Of 11 studies , 2 have identified de novo MECP2 mutations in female patients with ASD : a de novo splice variant in intron 2 ( IVS2 + 2 delTAAG ) , a frameshift mutation ( 1157 del41 ) and a nonsense mutation ( R294X ) .48 49 Interestingly , the phenotype associated with this mutation includes MR but <negation>not epilepsy</negation> , microcephaly , stereotypies or any of the symptoms characteristic of Rett syndrome ; regression was present in one patient , but <negation>not in the other two.4 8 49 Another study identified two de novo mutations ( R133C and R453X</negation> ) in girls with ASD fulfilling the criteria for the preserved speech variant of Rett syndrome.5 0 The remaining eight studies were either entirely negative or reported missense , intronic or 3 ? -untranslated region variants potentially of functional interest ; either these were reported to be inherited from one of the parents or it was not specified whether they were inherited or de novo.5 1 ? 58 Overall , MECP2 mutations are rare in the autistic population , accounting for 0.8 ? 1.3% of cases in the female ASD population ( ie , 3/378 or 5/397 , respectively , depending whether patients from the Zappella et al study50 are included ( table 4 ) and <negation>not including subjects from the Shibayama et al53 study for which no male : female ratio is provided )</negation> . | Sentence break error |
| 26 | UBE3A | <negation>Further evidence of the global importance of UBE3A</negation> , other ubiquitin ligases and related UPS proteins in cognitive function has come in a very recent publication identifying these proteins along with neuronal adhesion proteins as exhibiting copy number variation in a cohort of over 800 patients suffering from autism spectrum disorders [ 36 ] . | Scope error |
| 27 | UBE3A | Based on evidence that maternal but <negation>not paternal duplications of chromosome 15 q cause autism</negation> , we attempted to test the hypothesis that autism involves oligogenic inheritance ( two or more loci ) and that the Angelman gene ( UBE3A ) , which encodes the E6 -AP ubiquitin ligase , is one of the contributing genes . |  |
| 28 | UBE3A | UBE3A mutations due to <negation>lack of the homologous to the E6 -associated protein carboxyl terminus domain ( n = 3 ) were associated with the ASD</negation> , while more distal mutations ( n = 3 ) seem to escape from a co-morbid diagnosis of autism / autism spectrum . |  |
| 29 | RELN | Until recently , human studies have <negation>failed to reach consensus on the association of RELN gene and autism</negation> . |  |
| 30 | RELN | The rather small number of cases with these presumably functional mutations makes further analysis difficult , but is suggestive that RELN is involved in autism , though the authors conclude that this set of variants could <negation>not account for their previously published large linkage finding to 7 q22 ( International Molecular Genetic Study of Autism Consortium , 1998 , 2001 a , b )</negation> . |  |
| 31 | RELN | The Reelin gene ( approved gene symbol RELN ) locus maps to chromosome 7 q22 between D7S658 ( 113.92 cM ) and D7S1532 ( 113.92 cM ) on the Marshfield map , in proximity to or <negation>within the portion of chromosome 7 q yielding positive lod scores in most autism linkage studies published to date</negation> . |  |
