# Supplementary material for: BioN∅T: A searchable database of biomedical negated sentences
Source: BMC Bioinformatics. 2011 Oct 27;12:420. doi: 10.1186/1471-2105-12-420 (PMC3225379; doi:10.1186/1471-2105-12-420)
Supplement: Additional file 2 — Ambiguous negated association between the gene and disease. This file lists the false positive associations caused because the negated association between the gene and disease was ambiguous. [file 1471-2105-12-420-S2.DOCX]

Additional File 2: Ambiguous negated association between the gene and disease

| Count | Gene | Sentence | Notes |
| --- | --- | --- | --- |
| 1 | NRXN1 | A scan of the NRXN1 coding sequence in a cohort of ASD subjects , relative to non-ASD controls , revealed that amino acid alterations in neurexin 1 are <negation>not present at high frequency in ASD</negation> . |  |
| 2 | CNTNAP2 | It should be noted , however , that although this idea is currently popular , it is not fully supported by behavioral data on relatives ; the broad phenotype of autism does <negation>not appear to encompass the kind of nonword repetition deficits associated with CNTNAP2</negation> and seen in individuals with SLI and their relatives ( Bishop et al. 2004 ; Whitehouse et al. 2007 ) . |  |
| 3 | MET | In contrast , there was <negation>no association of the MET polymorphism with autism spectrum disorder in the 96 families lacking a child with co-occurring autism spectrum disorder and gastrointestinal conditions</negation> . |  |
| 4 | OXTR | Although the two SNPs might <negation>not play a prominent role in the functioning of the gene , the genetic contribution of OXTR to autism cannot be ruled out</negation> . |  |
| 5 | OXTR | The subsequent analysis of a group of unrelated autistic subjects did <negation>not show an OXTR deletion</negation> , but <negation>rather hypermethylation of the gene promoter</negation> , with a reduced mRNA expression. |  |
| 6 | OXTR | Importantly , in keeping with the hypothesis that increases in DNA methylation ( hypermethylation ) lead to gene silencing , CpG sites -934 and -924 showed the largest increase in methylation , 37.5% , between the autistic sibling and the non-autistic father ( 62.5% methylated versus 25% methylated , 100% methylated versus 62.5% methylated , respectively ) , both <negation>lacking the OXTR deletion</negation> . |  |
| 7 | SLC6A4 | Although genome-wide scans in autism provided support for linkage at SLC6A4 , TDT studies in autism have <negation>failed to reveal any consistent evidence of allele or haplotype association at SLC6A4</negation> . |  |
| 8 | NLGN3 | First , at the time of writing NLGN3 and NLGN4 possessed <negation>no annotation IDs related to their roles in autism and could both be identified within Ensembl ( see Materials and methods )</negation> . |  |
| 9 | PTEN | However , there <negation>are aspects of ASD that are not recapitulated in the Pten mutants</negation> , caution Greer and Wynshaw-Boris . |  |
| 10 | GABRB3 | Transmission disequilibrium test revealed that , 183 bp long allele in GABRB3 gene was preferentially transmitted in families with ASD ( p = 0.025 ) , whereas a population-based case-control study , however , showed <negation>no association between ASD and GABRB3 microsatellite polymorphism</negation> . |  |
| 11 | MECP2 | To test the hypothesis that pathways affecting MeCP2 expression changes may be defective in RTT , autism and other neurodevelopmental disorders <negation>without MECP2 mutations</negation> , a high-throughput quantitation of MeCP2 expression was performed on a tissue microarray containing frontal cortex samples from 28 different patients with neurodevelopmental disorders and age-matched controls . |  |
| 12 | MECP2 | <negation>The relevance of the three remaining mutations towards the aetiology of autism could not be ruled out</negation> , although they were not localised within functional domains of MeCP2 and may be rare polymorphisms . |  |
| 13 | UBE3A | <negation>Although evidence for UBE3A mutations in autism was not found in this series</negation> , the hypothesis is compelling and needs further investigation . |  |
| 14 | UBE3A | A search for epigenetic abnormalities led to the discovery of a tissue-specific differentially methylated region ( DMR ) downstream of the UBE3A coding exons , but the region was <negation>not abnormal in autism lymphoblasts or brain samples</negation> . |  |
| 15 | RELN | CONCLUSION : Thus , we conclude that there is <negation>no main effect of APOE in our autism data set , nor is there any evidence for a joint effect of APOE with RELN</negation> . |  |
| 16 | RELN | Should we therefore conclude that RELN is <negation>not involved in autism</negation> ? |  |
| 17 | RELN | Thus , although the case-control and affected sib-pair findings did <negation>not support a role for RELN in susceptibility to ASD</negation> , the more powerful family-based association study demonstrated that RELN alleles with larger numbers of CGG repeats may play a role in the etiology of some cases of ASD , especially in children <negation>without delayed phrase speech</negation> . |  |
