# Supplementary material for: BioN∅T: A searchable database of biomedical negated sentences
Source: BMC Bioinformatics. 2011 Oct 27;12:420. doi: 10.1186/1471-2105-12-420 (PMC3225379; doi:10.1186/1471-2105-12-420)
Supplement: Additional file 3 — Ambiguous terms. This file lists the false positive associations caused because either the gene name or the disease name was ambiguous. [file 1471-2105-12-420-S3.DOCX]

Additional File 3: Ambiguous terms

| Count | Gene | Sentence | Notes |
| --- | --- | --- | --- |
| 1 | MET | Additionally , there was a trend for orienting speed to differ between diagnostic outcome subgroups ; children with confirmed diagnoses of autism at time of behavioral testing had larger orienting deficits than those who <negation>no longer met diagnostic criteria for autism</negation> . | Met used as an English word |
| 2 | MET | For the narrow phenotype , all sib-pairs ( n117 ) met the full ADHD criteria <negation>without autism</negation> . | Met used as an English word |
| 3 | MET | Oliver met criteria for pervasive developmental disorder ( he did <negation>not meet strict criteria for autism )</negation> . | Met used as an English word |
| 4 | MET | Autism was rigorously defined and <negation>no subjects met criteria for mental retardation</negation> . | Met used as an English word |
| 5 | MET | <negation>None of them met , or had ever met</negation> , the diagnostic criteria for autism . | Met used as an English word |
| 6 | MET | <negation>None met DSM-IV nor ADOS-G</negation> ( same administration procedure of the autism group was applied ) criteria for autism . | Met used as an English word |
| 7 | MET | While all children met ADOS / ADI-R criteria for autism prior to treatment , <negation>no child met criteria at follow-up</negation> . | Met used as an English word |
| 8 | MET | Children were included in this sample who : ( 1 ) met full criteria for autistic disorder , ( 2 ) were no more than one point short of full criteria in the social domain and either the repetitive behavior or communication domain ( borderline autism ) , ( 3 ) did <negation>not meet full or borderline autism criteria</negation> but met DSM-IV criteria for Asperger 's Disorder , or ( 4 ) met onset criteria for autistic disorder but showed marginally sub-threshold deficits in either of the three core domains . | Met used as an English word |
| 9 | MET | Overall , 58% of surveyed psychiatrists and paediatricians indicated that , in the face of diagnostic uncertainty , they had erred on the side of providing an ASD diagnosis for educational ascertainment and 36% of clinicians had provided an autism diagnosis for Carer 's Allowance when Centrelink diagnostic specifications had <negation>not been met</negation> . | Met used as an English word |
| 10 | MET | Studies of psychosocial interventions for autism , anorexia / bulimia , post-traumatic stress disorder , bipolar disorder , obsessive ? compulsive disorder , panic disorder , and substance abuse had <negation>not yet met criteria for either of these categories</negation> . | Met used as an English word |
| 11 | MET | Our sample consisted of 50 sets of parents of disturbed children , 15 whose children had met Rimland 's stringent criteria for autism , 24 whose children , though <negation>not having met these criteria</negation> , had been diagnosed clinically as autistic , and 11 sets whose severely disturbed children were never diagnosed autistic . | Met used as an English word |
| 12 | MET | All ASD participants had their diagnosis confirmed by the Autism Diagnostic Observation Schedule ? Generic ( Lord et al. , 2000 ) , while <negation>no participant in the SLI or SLI-history group met criteria for ASD on this measure</negation> . | Met used as an English word |
| 13 | MET | All participants met the following criteria for SLI : ( 1 ) expressive and / or receptive language scores or composite 1.5 or more standard deviations below the mean ; ( 2 ) performance IQ of 80 or higher ; ( 3 ) <negation>no major neurologic abnormalities</negation> ; ( 4 ) hearing and corrected vision within normal limits ; ( 5 ) <negation>absence of known developmental disorders such as mental retardation or autism</negation> . | Met used as an English word |
| 14 | MET | Prior to inclusion in the present study , they underwent screening to insure that they met the following selection criteria : ( 1 ) performance IQ ( PIQ ) of 80 or higher on the WISC-R , WPPSI or Leiter non-verbal measures ; ( 2 ) <negation>no major neurological abnormalities ( determined by a neurological examination )</negation> ; ( 3 ) expressive language composite score 1.5 or more standard deviations below the mean using the CELF-R ( Semel , Wiig , & Secord , 1987 ) ; and ( 4 ) <negation>absence of known developmental disorders such as mental retardation or autism</negation> . | Met used as an English word |
| 15 | MET | They met the following selection criteria : ( 1 ) performance IQ ( PIQ ) of 80 or higher ; ( 2 ) <negation>no major neurologic abnormalities</negation> ; ( 3 ) expressive language composite score 1.5 or more standard deviations below the mean using the Clinical Evaluation of Language Fundamentals ? Revised ( CELF-R ) ( Semel , Wiig , & Secord , 1987 ) ; and ( 4 ) <negation>absence of known developmental disorders such as mental retardation or autism</negation> . | Met used as an English word |
| 16 | MET | Of these , 344 met criteria on both ADOS and ADI-R for either autism or ASD ( AU / ASD group ) , 68 were confirmed to have DD or atypical development <negation>without AU / ASD</negation> ( met criteria for DD on at least one of the MSEL and VABS ) and are designated DD , 166 recruited from the general population had confirmed typical development ( TD ) , and 40 did <negation>not fall into any of these diagnostic classes or had incomplete assessment data</negation> and were excluded from further analyses . | Met used as an English word |
| 17 | MET | Each family met the following inclusion criteria : the target child was between 2 and 6 years of age and had <negation>not yet started primary school</negation> ; the primary caregiver had one or more concerns about their child ? s behavior or their own parenting skills ; the child had <negation>not received a diagnosis of developmental delay</negation> , developmental disorder ( e.g. , autism ) , conduct disorder or ADHD ; the child was <negation>not currently taking medication or in regular contact with another professional for behavioral problems ; the parents were not currently in therapy for psychological or relationship problems</negation> ; and the parents were able to read English . | Met used as an English word |
| 18 | MET | Fifteen patients ( 45% ) diagnosed with ASD and 34 patients ( 7% ) <negation>not diagnosed with ASD subsequently met criteria for PTSD</negation> . | Met used as an English word |
| 19 | MET | At follow-up , PTSD was diagnosed in 53% of patients who had been diagnosed with ASD and in 11% of those who had <negation>not met criteria for ASD</negation> ; 36% of patients with PTSD did <negation>not initially display ASD</negation> . | Met used as an English word |
| 20 | MET | An additional 17 ( of 1,111 , 1.5% ) participants who did <negation>not meet criteria for ASD met criteria for PTSD at the 6 ? 10 week point</negation> . | Met used as an English word |
| 21 | MET | Six months later , 81.8% of ASD cases met criteria for PTSD , contrasting with 11.5% of those who did <negation>not develop ASD</negation> . | Met used as an English word. In this context, ASD stands for ‘acute stress disorder’ |
| 22 | MET | The majority of those who met criteria for subsyndromal ASD did <negation>not meet the ASD criteria for dissociation</negation> . | Met used as an English word |
| 23 | MET | In terms of those <negation>not diagnosed with ASD</negation> , 11% subsequently met criteria for PTSD . | Met used as an English word |
| 24 | MET | In terms of participants who participated in all 3 assessments , 63% who met the criteria for ASD , 70% who met the criteria for subsyndromal ASD , and 13% who did <negation>not meet the criteria for ASD</negation> were diagnosed with PTSD at 2 years posttrauma . | Met used as an English word |
| 25 | MET | Of the 41 participants that were evaluated and finished the study , 81% met the criteria for Autism , 12% more met the criteria for ASD , and 7% did <negation>not meet the criteria for ASD , according to the sum of the Communication + Social Scores</negation> . | Met used as an English word |
| 26 | MET | Results indicated that children with a previously documented ASD diagnosis had higher rates of autistic regression than children who met the ASD surveillance definition but did <negation>not have a clearly documented ASD diagnosis in their records</negation> ( 17-26 percent of surveillance cases ) . | Met used as an English word |
| 27 | MET | Participants were 27 individuals with high-functioning ( i.e. IQ ? 85 ) ASD ( 21 male and 6 female ) who met criteria for autistic disorder , Asperger 's disorder or pervasive developmental disorder <negation>not otherwise specified ( PDD-NOS ) in DSM-IV</negation> , 24 healthy non-affected siblings with ASD ( 11 male and 13 female ) , and 27 unrelated healthy controls with <negation>no family history of ASD</negation> ( 21 male and 8 female ) . | Met used as an English word |
| 28 | MET | A thromboembolic event was considered to be due to paradoxical embolism if the following criteria were met : ( 1 ) presence of PFO ( with or <negation>without septal aneurysm ) or ASD with spontaneous or provocable right-to-left</negation> shunt during contrast transesophageal echocardiography , ( 2 ) clinically and neurologically confirmed ischemic stroke or symptoms of transient ischemic attack with neuroradiologically identified intracranial ischemic or clinically and radiologically verified extracranial peripheral thromboembolism ; and ( 3 ) exclusion of any identifiable cause for the thromboembolic event other than the interatrial communication ( Table 1 ) . | Met used as an English word |
| 29 | MET | Children could be included in the ADHD group if ( 1 ) both the parent and teacher ratings were at or above the 95 th percentile on at least one of the two ADHD-related DBD scales to be sure that the pervasiveness criterion for ADHD ( DSM-IV-TR , 2000 ) was met ; ( 2 ) their estimated FSIQ was at or above 80 ; ( 3 ) the ADHD diagnosis was confirmed with the DISC-IV and there were <negation>no signs of OCD or TS</negation> ; ( 4 ) they had <negation>no characteristics of ASD as measured with the ADI-R</negation> . | Met used as an English word |
| 30 | MET | Autistic behavior had decreased in 19 percent such that they <negation>no longer met the criteria for autistic disorder</negation> . | Met used as an English word |
| 31 | MET | The group of children who , on followup , <negation>no longer</negation> met the diagnosis of autistic disorder had lower severity of illness ratings and higher intellectual functioning at baseline and at followup than those children who remained diagnosed as autistic disorder ; they also showed a rise in intellectual functioning over time whereas those children who remained diagnosed as autistic did <negation>not</negation> . | Met used as an English word |
| 32 | MET | Those children with autistic features who were <negation>not frankly autistic and would not have</negation> met DSM-IV criteria for pervasive developmental disorder were labeled suspect . | Met used as an English word |
| 33 | MET | The boys did <negation>not manifest autistic behavior</negation> , and none met criteria for an autism spectrum disorder , though their parents reported substantial repetitive behavior . | Met used as an English word |
| 34 | MET | Of the total group of 117 children evaluated in those families , 83 ( 71% ) met all ADI criteria and could be unambiguously classified as autistic ( affected ) , 26 ( 22% ) met <negation>none of the ADI criteria and were classified as not autistic ( unaffected )</negation> , and 8 ( 7% ) were classified as uncertain because they met one or more but <negation>not all of the ADI cutpoints</negation> . | Met used as an English word |
| 35 | MET | Senior pediatric neurologists evaluated all children at the time of admission to determine whether children met diagnostic criteria for autism or an autistic spectrum disorder ( ASD ) , or if they had isolated language regression ( language regression <negation>without autistic features )</negation> . | Met used as an English word |
| 36 | MET | Autistic ? traits " were also retrospectively found in almost one-quarter of 2201 adults previously diagnosed with various learning disabilities.2 5 Questionnaires devised to specifically diagnose ADHD will <negation>not identify autistic symptomatology</negation> , and 74% of children with high-functioning autism in another series had erroneously been previously diagnosed with ADHD despite clear differences in their social competence , cognitive development , and restricted range of activities. | Met used as an English word. Sentence break error. |
| 37 | MET | Five of 38 subjects who met DSM-IV criteria for autistic disorder at age 5 years <negation>no longer met criteria at their current age</negation> , although all five continued to have substantial impairment . | Met used as an English word |
